# Case report of a dissociative identity disorder

**DOI:** 10.1192/j.eurpsy.2021.1327

**Published:** 2021-08-13

**Authors:** R. Pinilla, C. Rodriguez, B. Ordoñez, R. Hermosillo

**Affiliations:** Psiquiatria, Hospital de Getafe, Madrid, Spain

## Abstract

**Introduction:**

Patients with dissociative identity disorder (DID) present two or more identities, where one of them is the main one. Although it is a widely questioned diagnosis, it is currently found in the main DSM-5 and ICD-10 diagnostic manuals.

**Objectives:**

Present a case of dissociative identity disorder.

**Methods:**

46-year-old woman who attended the CSM referred for her MAP due to anxiety-depressive symptoms. Throughout the interviews the patient brings up to 4 identities with alterations in memory, consciousness, multiple dissociative symptoms, sound thinking, constant fluctuations in mood. She is separated, has two children, takes care of them, although she is not able to maintain work functionality. The patient is seen once a week for 45 minutes. Psychotherapeutic treatment is carried out, the objective of which is to establish a safe therapist-patient bond to favor the integration of their parts, and pharmacological treatment, which was carried out with haloperidol, lorazepam and desvenlafaxine.

**Results:**

Throughout sessions, the anxious symptoms diminished, being able to carry out psychotherapeutic work. Dissociative symptoms were slightly reduced, partially integrating some of the identities. There was a slight stabilization in mood and decrease in psychotic symptoms.

**Conclusions:**

There is no well-established treatment for DID. Combined therapy (psychotherapy and pharmacological) may be an option for these patients. The therapeutic framing of the sessions, working the link, and the low-dose antipsychotic treatment were favorable.

**Keyword:**

dissociative identity framing link

